# Prevalence of *NRAS, PTEN* and *AKT1* gene mutations in the central nervous system metastases of non-small cell lung cancer

**DOI:** 10.1007/s10014-016-0276-2

**Published:** 2017-01-17

**Authors:** Marcin Nicoś, Paweł Krawczyk, Bożena Jarosz, Marek Sawicki, Tomasz Trojanowski, Janusz Milanowski

**Affiliations:** 1 Department of Pneumonology, Oncology and Allergology, Medical University of Lublin, Jaczewskiego 8, 20-954 Lublin, Poland; 2 Pathological Laboratory, Department of Neurosurgery and Pediatric Neurosurgery, Medical University of Lublin, 20-954 Lublin, Poland; 3 Department of Thoracic Surgery, Medical University of Lublin, 20-954 Lublin, Poland

**Keywords:** *AKT1*, *PTEN*, *NRAS*, NSCLC, Central nervous system metastases

## Abstract

Somatic mutations in *NRAS, PTEN* and *AKT1* genes are rarely (~1%) reported in primary NSCLC, but their role in carcinogenesis have been proven. Therefore, we assessed the frequency of them in 145 FFPE tissue samples from CNS metastases of NSCLC using the real-time PCR technique. We identified four (two *NRAS* and single *AKT1* and *PTEN*) mutations in CNS metastases of NSCLC. All mutations were observed in current male smokers (4% out of the male group; 4/100 and 4.25% out of smokers; 4/94). Three mutations have been detected in patients with SqCC (10.3% out of SqCC patients; 3/29), and only one mutation in the *NRAS* gene—in a patient with adenocarcinoma (1.25% out of AC patients; 1/80). The examined genes were mutually exclusive in terms of molecular background in *KRAS; EGFR; DDR2; PIK3CA; HER2* and *MEK1* genes that were evaluated in our previous studies. The OS of the patients who harbored *NRAS, AKT1* and *PTEN* mutations was 10.1, 12.1, 7.3 and 4 months, respectively (vs 13.5 months of the studied group). Our results suggest that the presence of *NRAS, PTEN* and *AKT1* gene mutations may have an influence on the occurrence of CNS metastases in patients with SqCC.

## Bacground

Among metastatic sites of non-small cell lung cancer (NSCLC) central nervous system (CNS) lesions occur in 20–40% of lung adenocarcinoma (AC) patients and they are associated with neurological symptoms and extremely poor survival prognosis. In squamous cell carcinoma (SqCC), CNS metastases are observed less frequently (10–15%) [1–4]. In patients with CNS metastases, the administration of standard chemotherapies or targeted agents is limited because of uncertain penetration of anticancer drugs through the blood–brain barrier and poor prognosis [5–8].

Today, we have found that mutational deregulations of pro-survival (PI3K-mTOR-AKT) and proliferative (Ras-Raf-Mek-Erk) cascades play a crucial role in uncontrolled signal transduction in cancer cells [9–11]. The majority of mutations involved in NSCLC carcinogenesis were reported in five oncogenes: *KRAS, EGFR, ALK, HER2* and *BRAF* [10, 12]. However, the presence of mutations in *NRAS, MEK, AKT1, PTEN, RET, ROS1* genes had identified impact on acquiring resistance to both EGFR or ALK TKIs and radiotherapy [9, 13]. Some papers reported that the MEK (selumetinib and trametinib), IGF-1R (linsitinib) or allosteric PI3K (LY294002) inhibitors may become an attractive therapeutic choice for NSCLC patients with some rare mutations [4, 9–11, 14–16].

To date, the majority of published data evaluated gene mutations in primary tumors of NSCLC. However, there is still limited data assessing genetic disorders in metastatic lesions. In our previous study, we focused on commonly mutated genes playing role in carcinogenesis. Therefore, the following study, we evaluated the prevalence of *NRAS, PTEN* and *AKT1* gene mutations in Caucasian patients with CNS metastases of NSCLC.

## Methods

### Patients

The studied group included 145 Polish NSCLC patients with CNS metastases of advanced NSCLC. In 30 patients, the corresponding primary tumors were simultaneously available. The patients underwent routine neurosurgical procedures with a palliative manner. In the moment of CNS metastases diagnosis, all patients were chemo-, radio- or targeted therapy naïve. They did not receive any other treatment which could affect mutation inducement. The median overall survival (OS) was 13.5 months (range 0.1–78.2 months—information available from 119 patients). Detailed characteristics of the studied group have been presented in Table 1. DNA was isolated from formalin-fixed paraffin-embedded (FFPE) tissue samples using the QIAamp DNA FFPE Tissue Kit (Qiagen, USA) according to manufacturer protocol. The positive control of the analysis was the reaction with control DNA supplied with the assay by the manufacturer. DNA isolated from peripheral blood leukocytes of healthy individuals (*n* = 10) provide the negative control of analysis. The study was approved by the Ethics Committee of the Medical University of Lublin, Poland (No. KE-0254/86/2013). All patients expressed their consent to participate in the study and they expressed their consent to publish their individual data (if it is needed).

**Table 1:** Detailed studied group characteristics

Gender	
Male, *n* (%)	100 (69)
Female, *n* (%)	45 (31)
Age	
Median age ± SD (years)	60 ± 8.8
≥60 years, *n* (%)	72 (49.7)
<60 years, *n* (%)	73 (50.3)
Histopathology	
Adenocarcinoma, *n* (%)	80 (55.2)
Squamous cell carcinoma, *n* (%)	29 (20)
Large-cell carcinoma, *n* (%)	22 (15.1)
NSCLC-NOS, *n* (%)	14 (9.7)
Smoking status	
Current smokers, *n* (%)	73 (50.4)
Former smokers, *n* (%)	21 (14.5)
Non-smokers, *n* (%)	36 (24.8)
Lack of data, *n* (%)	15 (10.3)
Performance status (ECOG/WHO)	
0, *n* (%)	22 (15.2)
1, *n* (%)	76 (52.4)
2, *n* (%)	31 (21.4)
3, *n* (%)	16 (11)

### NRAS mutation analysis

*NRAS* gene status was evaluated using the *NRAS* Mutation Analysis Kit (EntroGen, USA) certified for *in vitro* diagnosis (CE-IVD). This kit determines 12 substitutions (G12D, G12S, G12C, G13R, G13V, Q61K, Q61L, Q61R, Q61H, A126T, K117R, A59X) located in exons 2, 3 and 4 of the *NRAS* gene. The amplification of the examined region was performed in 96-well plates in a real-time PCR device (Cobas, Roche, USA) in the following steps: pre-denaturation in 95 °C for 10 min. and 40 cycles in conditions: 95 °C for 15 s. and 60 °C for 40 s.

### PTEN and AKT1 mutation analysis

*PTEN* and *AKT1* gene mutations were evaluated using the TaqMan Mutation Detection Assay (Applied Biosystem, USA) certified for research use. The amplification of examined genes was performed in 96-well plates in a real-time PCR device (Cobas, Roche, USA) in the steps recommended by Applied Biosystems: 95 °C for 10 min and 92 °C for 15 s, 58 °C for 1 min. for five cycles then 92 °C for 15 s, 60 °C for 60 s for 40 cycles.

### Results analysis

Fluorescence was observed only during amplification of mutant types (mt) in analyzed samples and in the endogenous control. According to observations made of the positive and negative control amplification plots, samples were assessed as mt if we observed amplification between 25 and 30 cycles. The samples without or with late amplification (*C*_*t*_ > 35 cycle) were assessed as wild type (wt). Based on amplification curves in mt samples and the corresponding endogenous wt control, we estimated the frequency of mt DNA according to the following equation:

$$\%\text{ mutated DNA}={{\text{2}}^{-\Delta Ct}}\, \times \, \text{100}\%$$

Δ*Ct* (analyzed sample) = the average *Ct* value from the mutant reaction—the average *Ct* value from the wild-type reaction.

## Results

**Fig. 1:**
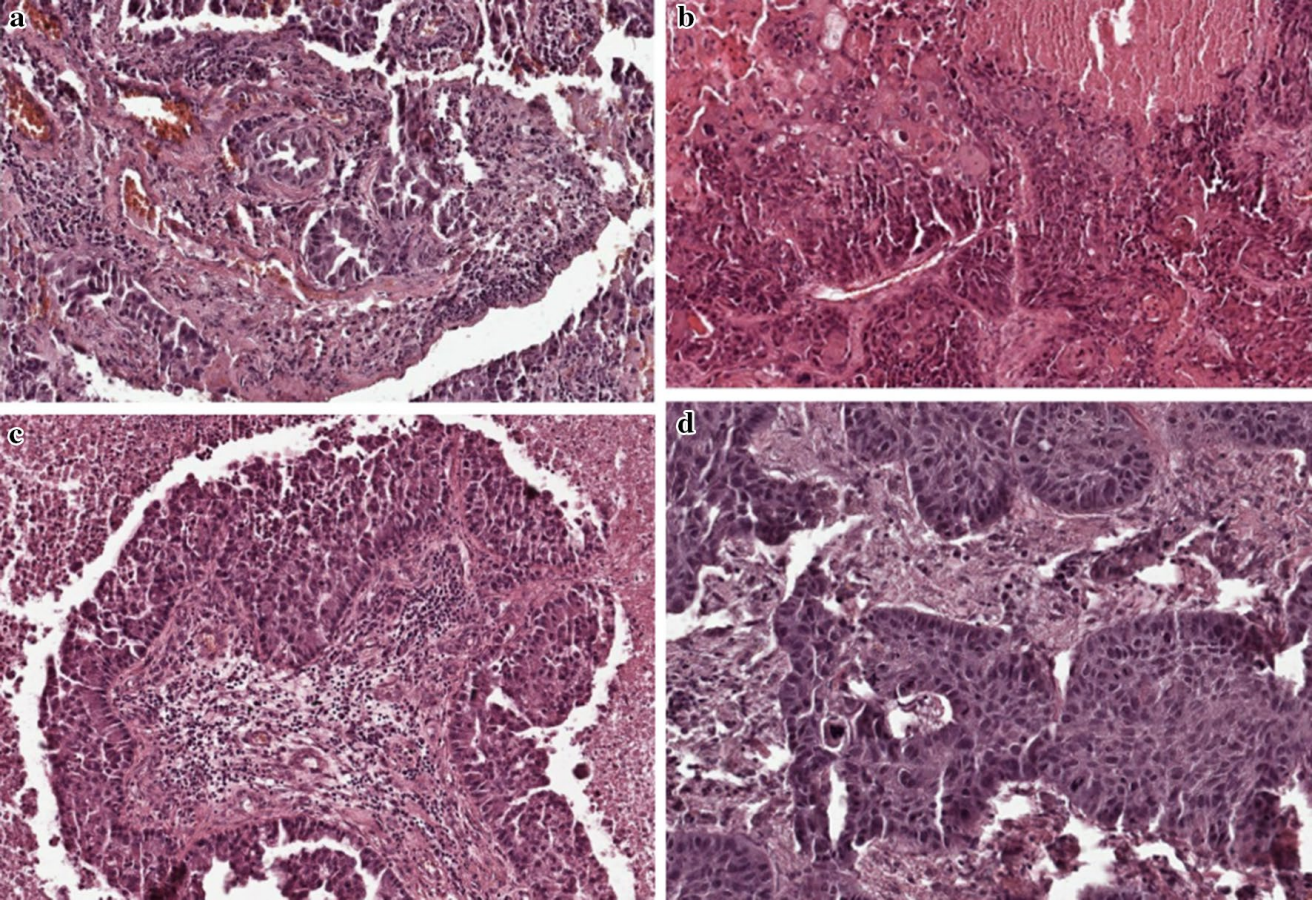
Slides presenting histopathology differentiation of patients with detected mutations. **a** Shows adenocarcinoma type of NSCLC in patients with Q61L substitution in *NRAS* gene. **b** Shows squamous cell carcinoma type of NSCLC in patients with A126T substitution in *NRAS* gene. **c** Shows squamous cell carcinoma type of NSCLC in patients with E17K substitution in *AKT1* gene. **d** Shows squamous cell carcinoma type of NSCLC in patients with R233X substitution in *PTEN* gene

We identified four (two *NRAS* and single *AKT1* and *PTEN*) mutations in CNS metastases of NSCLC. The content of mt allele in all mutated samples was >5%. All mutations were observed in current male smokers (4% out of the male group; 4/100 and 4.25% out of smokers; 4/94). Three mutations have been detected in patients with SqCC (10.3% out of SqCC patients; 3/29), and only one mutation in the *NRAS* gene—in a patient with adenocarcinoma (1.25% out of AC patients; 1/80). Slides presenting histopathology differentiation for patients with detected mutation were presented at Fig. 1. The examined genes were mutually exclusive in terms of molecular background in *KRAS; EGFR; DDR2; PIK3CA; HER2* and *MEK1* genes that were evaluated in our previous studies [17–22]. A simultaneous evaluation of 30 patients in whom both CNS metastases and the corresponding primary tumors were available, showed the presence of wt in *NRAS, AKT1* and *PTEN* genes in both lesions. Unfortunately, the corresponding primary tumors were unavailable from patients who harbored *NRAS, AKT1* and *PTEN* mutations in CNS metastases. Due to low quality of DNA and sub-clonality of CNS metastases we did not perform deep sequencing approaches to confirm our results.

Using an EntoGen kit we identified Q61L and A126T substitutions in the *NRAS* gene in two patients (1.4% out of all patients; 2/145). A Q61L substitution was observed in a 47-year-old patient (35 pack-years) with AC histology (1.25% out of AC patients; 1/80); while an A126T substitution was found in a 71-year-old patient (20 pack-years) with squamous cell carcinoma histology (3.5% out of SqCC patients; 1/29). The overall survival (OS) of *NRAS* mutated patients was 10.1 and 12.1 months, respectively.

Using TaqMan hydrolysis probes we detected an E17K substitution in the *AKT1* gene (0.7% of studied group) and an R233X substitution in the *PTEN* gene (0.7% of the studied group). The *AKT1* gene mutation was observed in a 73-year-old patient (20 pack-years) with SqCC histology (3.5% out of SqCC patients; 1/29). The *PTEN* gene mutation was found in a 62-year-old patient (50 pack-years) with SqCC histology (3.5% out of SqCC patients; 1/29). The OS of *AKT1* and *PTEN* mutated patients was 7.3 and 4 months, respectively.

The summary of clinical and demographical data of positive patients has been presented in Table 2.

**Table 2:** The summary of clinical and demographic data of NSCLC patients with *NRAS, AKT1* and *PTEN* gene mutations

Gene	Substitution	Histology	Age	Gender	Smoking (pack-years)	OS (months)
NRAS	Q61L	AC	47	M	Former (35)	10.1
	A126T	SqCC	71	M	Former (20)	12.1
AKT1	E17K	SqCC	73	M	Former (20)	7.3
PTEN	R233X	SqCC	62	M	Current (50)	4

## Discussion

Brain metastases are one of the most common metastatic lesions of NSCLC which are associated with a high mortality of patients [1–4]. Moreover, a blood–brain barrier ensures restrict transit of agents into the brain parenchyma, which are considered as pharmacological sanctuary lesions that show limited sensitivity to anti-cancer therapy [1, 2]. However, there are some studies which indicated the effectiveness of anti-ALK targeted therapies (alectinib and ceritinib) also in CNS metastatic sites of NSCLC [6, 23]. Till today, we have only limited data concerning the evaluation of the most frequent mutations in *EGFR, KRAS, BRAF* genes in CNS metastases of lung cancer (especially AC type). Despite *NRAS, PTEN* and *AKT1* mutations have proven involvement in carcinogenesis [24–28], their frequency was described in a few reports only in primary NSCLC tumors [3, 4, 13, 14, 16]. Therefore, we performed the current and unique characteristic of the incidence of *NRAS, AKT1* and *PTEN* gene mutations in CNS metastases of NSCLC.

### NRAS gene mutations in NSCLC patients

NRAS as a member of the RAS family plays a role in the MAPK signaling pathway and its deregulation can lead to tumorgenesis [14, 29, 30]. Activating mutations in exons 2 (codons 12 and 13), 3 (codons 59 and 61) and 4 (codons 117, 126 and 146) of the *NRAS* gene have been frequently described in melanoma (13–25%), myeloid leukemia (10%), colorectal cancer (2–5%), hepatocellular carcinoma (1.4%) and thyroid carcinoma (6%) [31]. Among all well-known *NRAS* activating mutations, the substitutions in codon 61 are more frequent (90%) than substitutions in other codons [10, 14, 30]. The most common transversions are described as G > C and T > A. It was previously reported that air fossil fuel pollution (including di-methylo-benza-anthracene) are involved in the induction of A > T and T > A changes. Moreover, the combination of smoking and environmental carcinogens can be associated with the etiology of *NRAS* mutated lung cancer [14, 32].

In our analysis, we identified two *NRAS* mutations in CNS metastases of NSCLC including one Q61L substitution, which is reported in the literature as the most frequent type [10, 14, 30, 31]. The A126T substitution was the second *NRAS* mutation which is described as extremely rare [10, 14, 30]. To date, only Preusser et al. identified one (1.3%; 1/76) *NRAS* mutation in brain metastases of lung AC [4]. Most of comprehensive analyses that were carried out in the primary NSCLC, reported an extremely rare frequency (<1%) of *NRAS* mutation and described them as related to AC type and current smoking status [12, 14, 28]. Moreover, Ohashi et al. observed overlapping of one *NRAS* mutation with *KRAS* G12A substitution and another one with *MET* gene amplification [12, 14, 24].

### AKT1 gene mutation in NSCLC patients

AKT1 promotes the PI3K signaling pathway and is involved in cells proliferation and motility [9, 29]. A main E17K substitution in the *AKT1* gene was first identified by Carpten et al. in 2007 in breast cancers (8%), and to date, it was also reported in other solid tumors: colorectal (6%) and bladder cancers (5%) [10, 25].

The following analysis is the first report worldwide that describes one E17K substitution in the *AKT1* gene in CNS metastases of NSCLC. The corresponding primary tumor from this patient showed a native form of the *AKT1* gene. It indicates that the molecular status of the *AKT1* gene could be changeable during disease progression and disturbances in the PI3K-mTOR-AKT pathway can be involved in the process of metastasis [3, 4, 9]. The frequency of *AKT1* mutation in primary NSCLC tumors is also reported as low (<1%). These mutations occur more frequently in smokers and in SqCC [10, 12, 16, 24, 26, 27, 33–35]. However, Malanga et al. described the higher frequency of *AKT1* mutation (1.9%; 2/105) in NSCLC patients, especially in smokers and in SqCC type (5.5%; 2/36). They also suggested that hyperactivation of AKT1 cascade (due to E17K mutation) may be involved in the development of SqCC tumors [34].

### PTEN gene mutation in NSCLC patients

Phosphatase and tensin homolog (*PTEN*) is described as a tumor suppressor gene whose product regulates a PI3K-AKT-mTOR signaling cascade. The PTEN is involved in the stimulation of apoptosis, inhibition of cells’ migration and regulation in both p53 protein levels and activity [11, 13, 28]. The most common *PTEN* abnormality was described as a loss of heterozygosity and promoter methylation of the PTEN gene (~50% of NSCLC patients), however, some data also reported the presence of inactivating *PTEN* mutations [28, 36, 37].

In our analysis, we identified one inactivating *PTEN* gene mutation in CNS metastases of NSCLC and it is lower than *PTEN* mutation incidence reported in primary NSCLC (~4%). The previous literature data concerned the frequency of all inactivating *PTEN* mutations that were identified in NSCLC patients [28, 36]. However, in the following study we focused on only one mutation (R233X substitution) for which there are some indications of its clinical significance [11, 15, 28, 36, 37]. Jin et al. and Lee et al. showed that in the primary NSCLC, *PTEN* mutations are related to smoking and SqCC types [28, 36], however, significant relations to other clinicopathologic factors, such as age, gender and degree of cell differentiation have not been reported [28, 36, 37]. Some studies also indicated overlapping of *PTEN* mutations with *EGFR; ERBB2; KRAS* and *TP53* mutations [15, 28, 37]. In our study, all examined genes were mutually exclusive from *KRAS; EGFR; DDR2; PIK3CA; NRAS; HER2* and *MEK1* genes.

## Conclusions

In summary, particular *NRAS, AKT1* and *PTEN* gene mutations occur with similar rare (~1%) frequency in CNS metastases of NSCLC as in primary lung cancer tumors. Identification of the mutations is more likely in SqCC patients (especially in male smokers). Our results suggest that it is most likely to indicate the occurrence of *NRAS, AKT1* and *PTEN* mutations in metastatic sites of squamous cell lung carcinoma. An evaluation of the effectiveness of molecularly targeted agents in patients who harbor the mutations might be considered as a beneficial therapeutic choice in NSCLC patients with CNS metastases.

## Abbreviations

AC: Adenocarcinoma
CE-IVD: Certified for *in vitro* diagnosis
CNS: Central nervous system
FFPE: Formalin-fixed paraffin-embedded
NSCLC: Non-small cell lung cancer
mt: Mutant types
OS: Overall survival
SqCC: Squamous cell carcinoma
TKIs: Tyrosine kinase inhibitors
wt: Wild type

## References

[1] Renfrow JJ, Lesser GJ (2013). Molecular subtyping of brain metastases and implications for therapy. Curr Treat Options Oncol.

[2] Takei H, Rouah E, Ishida Y (2015). Brain metastasis: clinical characteristics, pathological findings and molecular subtyping for therapeutic implications. Brain Tumor Pathol.

[3] Villalva C, Duranton-Tanneur V, Guilloteau K (2013). EGFR, KRAS, BRAF, and HER-2 molecular status in brain metastases from 77 NSCLC patients. Cancer Med.

[4] Preusser M, Berghoff AS, Koller R (2015). Spectrum of gene mutations detected by next generation exome sequencing in brain metastases of lung adenocarcinoma. Eur J Cancer.

[5] Welsh JW, Komaki R, Amini A (2013). Phase II trial of erlotinib plus concurrent whole-brain radiation therapy for patients with brain metastases from non-small-cell lung cancer. J Clin Oncol.

[6] Gadgeel SM, Gandhi L, Riely GJ (2014). Safety and activity of alectinib against systemic disease and brain metastases in patients with crizotinib-resistant ALK-rearranged non-small-cell lung cancer (AF-002JG): results from the dose-finding portion of a phase 1/2 study. Lancet Oncol.

[7] Kaneda H, Okamoto I, Nakagawa K (2013). Rapid response of brain metastasis to crizotinib in a patient with ALK rearrangement positive non-small-cell lung cancer. J Thorac Oncol.

[8] Robinson SD, O’Shaughnessy JA, Cowey CL (2014). BRAF V600E-mutated lung adenocarcinoma with metastases to the brain responding to treatment with vemurafenib. Lung Cancer.

[9] Vijayalakshmi R, Krishnamurthy A (2011). Targetable “driver” mutations in non small cell lung cancer. Indian. J Surg Oncol.

[10] Greulich H (2010). The genomics of lung adenocarcinoma: opportunities for targeted therapies. Genes Cancer.

[11] Nurwidya F, Takahashi F, Murakami A (2014). Acquired resistance of non-small cell lung cancer to epidermal growth factor receptor tyrosine kinase inhibitors. Resp Invest.

[12] Kris MG, Johnson BE, Berry LD (2014). Using multiplexed assays of oncogenic drivers in lung cancers to select targeted drugs. JAMA.

[13] Huang L, Fu L (2015). Mechanisms of resistance to EGFR tyrosine kinase inhibitors. Acta Pharm Sin B.

[14] Ohashi K, Sequist LV, Arcila ME (2013). Characteristics of lung cancers harboring NRAS mutations. Clin Cancer Res.

[15] Su J, Zhang XC, An SJ (2014). Detecting the spectrum of multigene mutations in non-small cell lung cancer by Snapshot assay. Chin J Cancer.

[16] Do H, Solomon B, Mitchell PL (2008). A. Detection of the transforming AKT1 mutation E17K in non-small cell lung cancer by high resolution melting. BMC Res Notes.

[17] Kamila WK, Michał S, Paweł K (2013). EGFR activating mutations detected by different PCR techniques in Caucasian NSCLC patients with CNS metastases: short report. Clin Exp Metastasis.

[18] Nicoś M, Krawczyk P, Jarosz B (2016). Analysis of KRAS and BRAF genes mutation in the central nervous system metastases of non-small cell lung cancer. Clin Exp Med.

[19] Nicoś M, Powrózek T, Krawczyk P (2014). Sensitive methods for detection of the S768R substitution in exon 18 of the DDR2 gene in patients with central nervous system metastases of non-small cell lung cancer. Med Oncol.

[20] Nicoś M, Krawczyk P, Jarosz B (2016). Sensitive methods for screening of the MEK1 gene mutations in patients with central nervous system metastases of non-small cell lung cancer. Clin Transl Oncol.

[21] Krawczyk P, Nicoś M, Powrózek T (2013). Sensitive methods for the detection of an insertion in exon 20 of the HER2 gene in the metastasis of non-small cell lung cancer to the central nervous system. Oncol Lett.

[22] Nicoś M, Krawczyk P, Powrózek T (2016). PIK3CA mutations detected in patients with central nervous system metastases of non-small cell lung cancer. Anticancer Res.

[23] Shaw AT, Kim DW, Mehra R (2014). Ceritinib in ALK-rearranged non-small-cell lung cancer. N Engl J Med.

[24] Cancer Genome Atlas Research Network (2014). Comprehensive molecular profiling of lung adenocarcinoma. Nature.

[25] Carpten JD, Faber AL, Horn C (2007). A transforming mutation in the pleckstrin homology domain of AKT1 in cancer. Nature.

[26] Kim MS, Jeong EG, Yoo NJ (2008). Mutational analysis of oncogenic AKT E17K mutation in common solid cancers and acute leukaemias. Br J Cancer.

[27] Bleeker FE, Felicioni L, Buttitta F (2008). AKT1(E17K) in human solid tumours. Oncogene.

[28] Jin G, Kim MJ, Jeon HS (2010). PTEN mutations and relationship to EGFR, ERBB2, KRAS, and TP53 mutations in non-small cell lung cancers. Cancer.

[29] Shimizu K, Goldfarb M, Suard Y (1983). Three human transforming genes are related to the viral ras oncogenes. Proc Natl Acad Sci USA.

[30] Ding L, Getz G, Wheeler DA (2008). Somatic mutations affect key pathways in lung adenocarcinoma. Nature.

[31] Vujic I, Posch C, Sanlorenzo M (2014). Mutant NRASQ61 shares signaling similarities across various cancer types–potential implications for future therapies. Oncotarget.

[32] Osaka M, Matsuo S, Koh T (1995). N-ras mutation in 7,12-dimethylbenz[a]anthracene (DMBA)-induced erythroleukemia in Long-Evans rats. Cancer Lett.

[33] Pao W, Girard N (2011). New driver mutations in non-small-cell lung cancer. Lancet Oncol.

[34] Malanga D, Scrima M, De Marco C, Fabiani F, De Rosa N (2008). Activating E17K mutation in the gene encoding the protein kinase AKT1 in a subset of squamous cell carcinoma of the lung. Cell Cycle.

[35] Cancer Genome Atlas Research Network (2012). Comprehensive genomic characterization of squamous cell lung cancers. Nature.

[36] Lee SY, Kim MJ, Jin G (2010). Somatic mutations in epidermal growth factor receptor signaling pathway genes in non-small cell lung cancers. J Thorac Oncol.

[37] Malkoski SP, Cleaver TG, Thompson JJ (2014). Role of PTEN in basal cell derived lung carcinogenesis. Mol Carcinog.

